# Acute non-traumatic subdural hematoma induced by intracranial aneurysm rupture: A case report and systematic review of the literature

**DOI:** 10.1097/MD.0000000000021434

**Published:** 2020-07-31

**Authors:** Xianfeng Gao, Fagui Yue, Fenglei Zhang, Yang Sun, Yang Zhang, Xiaobo Zhu, Wei Wang

**Affiliations:** a Department of Neurosurgery; b Center for Reproductive Medicine and Center for Prenatal Diagnosis; c Department of Imaging, First Hospital, Jilin University, Changchun, China.

**Keywords:** aneurysm, non-traumatic, spontaneous subdural hematoma

## Abstract

**Rationale:**

Intracranial aneurysm with the first manifestation of acute subdural hematoma (aSDH) is rare in the field of neurosurgery. Usually subarachnoid hemorrhage or intracranial hematoma happens after the rupture of an intracranial aneurysm, whereas trauma is the primary cause of aSDH.

**Patient concerns:**

Here, we present the case of a 71-year-old woman who presented with spontaneous aSDH with progressive headache and vomiting.

**Diagnosis:**

Urgent head computed tomography (CT) identified an aSHD, but the patient had no history of trauma. CT angiography (CTA) identified the cause of the aSDH as rupture of an intracranial aneurysm in the left middle cerebral artery.

**Interventions:**

Emergent craniotomy with hematoma evacuation was performed.

**Outcomes:**

Due to prompt diagnosis and appropriate intervention, the patient recovered fully with no disability.

**Lessons:**

This unique case demonstrates that aSDH caused by intracranial aneurysm rupture requires timely identification and appropriate action to prevent adverse outcomes. We performed a comprehensive systematic literature review to examine the etiology and pathogenesis of non-traumatic aSDH. Furthermore, digital subtraction angiography should be considered in patients diagnosed with an aSDH with no known cause.

## 1 Introduction

Although spontaneous acute subdural hematoma (aSDH) in the absence of trauma is a rare condition,^[1]^ the associated mortality and morbidity rates are high. Non-traumatic aSDH is far less common that spontaneous aSDH occurring in the absence of an intracranial aneurysm. The etiology and pathogenesis remain uncertain given the infrequency of spontaneous non-traumatic aSDH. Arteriovenous fistulas,^[2]^ rupture of an arachnoid cyst^[3,4]^ or vasculature structure, hematological malignancies,^[5]^ coagulation defects,^[6–8]^ and cocaine abuse^[9]^ have been reported to contribute to the occurrence of non-traumatic aSDH in specific cases. In particular, the rupture of an intracranial aneurysm may have severe consequences if misdiagnosed, with an associated mortality estimated to range from 60% to 76.5%.^[10]^ Typical clinical manifestations are symptoms caused by intracranial hypertension, such as vomiting, headache, conscious disturbance, visual impairment, and brain hernia. Treatments include surgical decompression, hematoma evacuation, ventricular drainage, and conservative therapy.

In the report, we present a case of aSDH in a patient who presented with progressive headache and vomiting but no history of recent trauma. In consideration of the low incidence of the condition, the current literature was reviewed to elucidate the etiology and pathogenesis of non-traumatic aSDH as well as current standards for its diagnosis, treatment, and prognosis.

## 2 Case report

**Figure 1:**
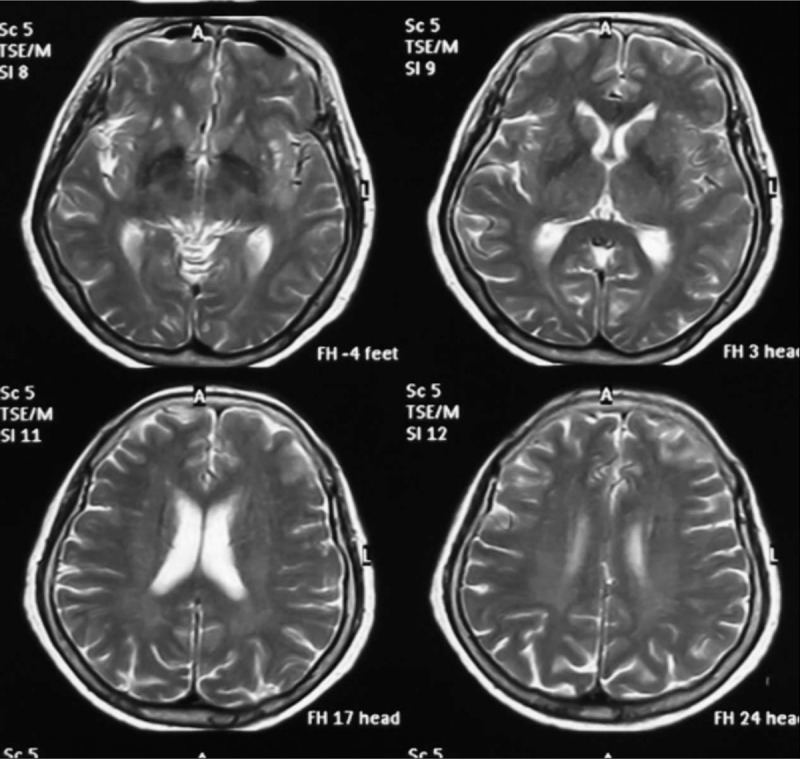
Magnetic resource imaging scans taken 1 month before hospitalization.

**Figure 2:**
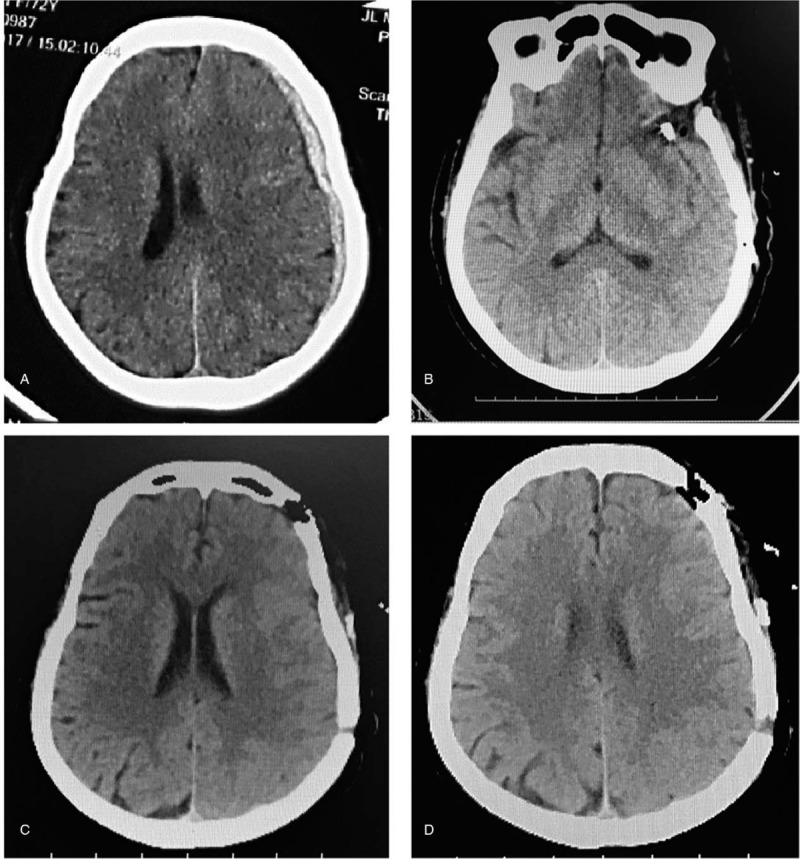
Computed tomography scans taken before surgery (A) and 2 days after surgery (B–D).

**Figure 3:**
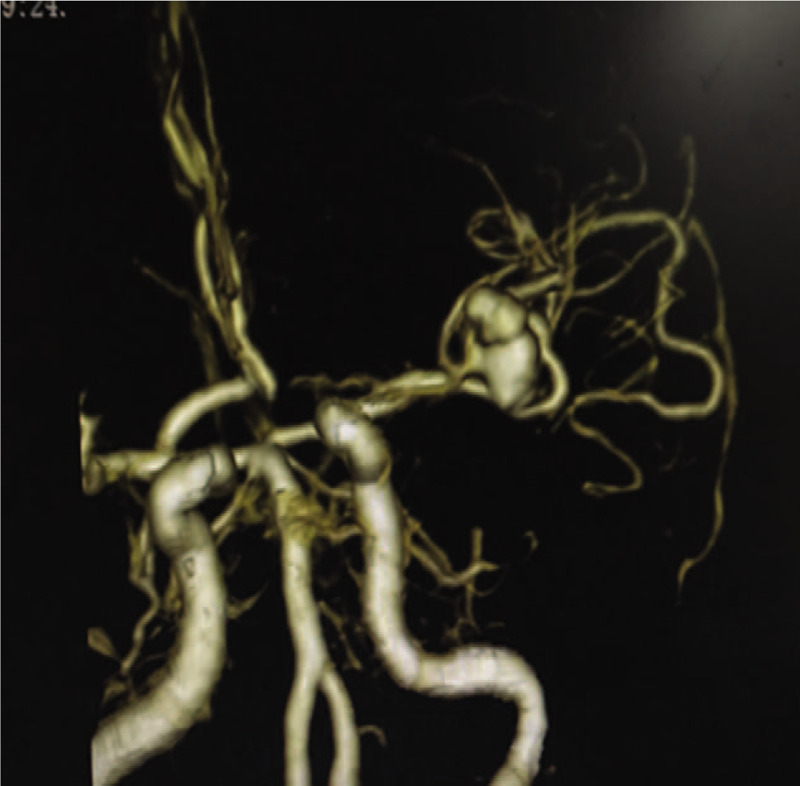
Computed tomography angiography scan showing aneurysm found in the left middle cerebral artery.

A 71-year-old woman presented to the out-patient department with progressive headache and vomiting without a recent history of traumatic injury. The patient had no history of hypertension and medication use. She complained of a sudden headache occurring 2 weeks previously as well as occurring approximately 6 weeks previously. Two weeks prior to her presentation at our hospital, she visited a local hospital for computed tomography (CT) and magnetic resource imaging (MRI) examinations with no positive results (Fig. 1). Symptom-based treatments were given to her at that time. On examination in our hospital, CT showed the patient had aSDHs in the left temporal lobe with central line deviation (Fig. 2). The initial laboratory tests yielded no abnormal results. Given the unknown cause of non-traumatic aSDH, magnetic resonance angiography (MRA), and computed tomography angiography (CTA) were performed, and an intracranial aneurysm was found in the M1 distal bifurcation of the left middle cerebral artery (Fig. 3).

**Figure 4:**
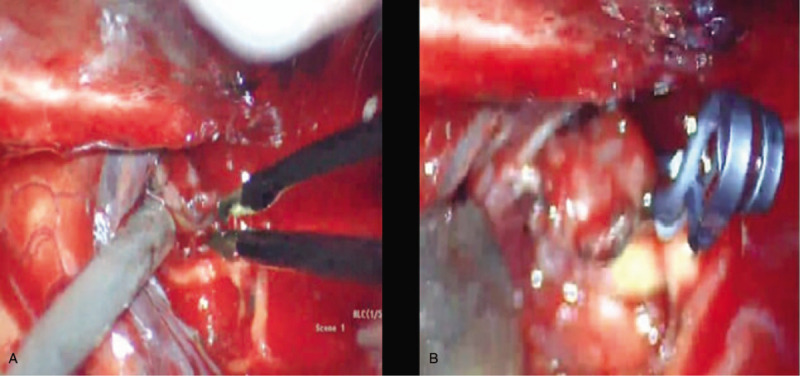
Intraoperative view showing aneurysm adhesion (A) and clipping (B).

Emergent craniotomy with hematoma evacuation was performed in the left brain. The intracranial aneurysm in the left middle cerebral artery was clipped in the operation. Obvious adhesions were observed intraoperatively between the aneurysm and arachnoid membrane (Fig. 4). The patient experienced a full recovery (Fig. 2) and was discharged 2 weeks later with a Glasgow outcome scale of 5. The Ethics Committee of the First Hospital of Jilin University approved our study protocol, and the patient had provided informed consent for publication of the case. (NO. is 2019–296).

## 3 Discussion

**Figure 5:**
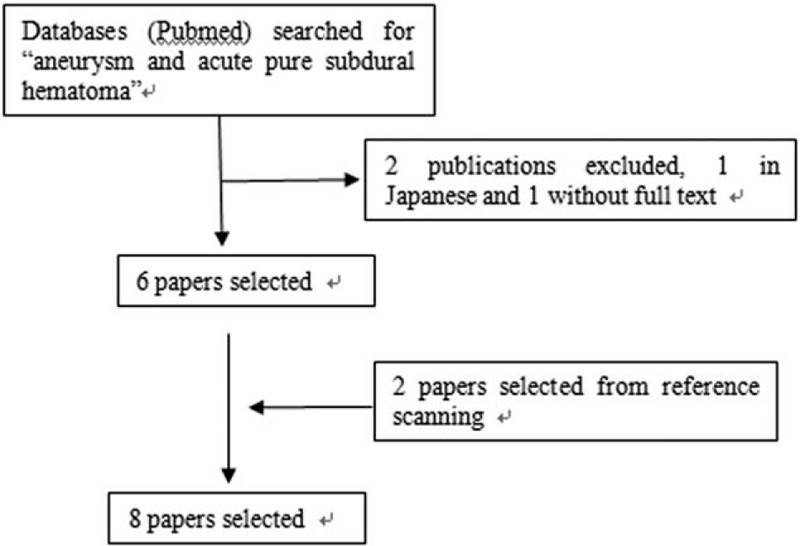
Flow chart of the review of literature.

A review of English reports of aSDH case caused by aneurysm was conducted by searching the PubMed databased between January 2013 and January 2018. The terms “aneurysm and acute pure subdural hematoma” were used to search for the publications. Eight publications were eventually included in our analysis (Table 1). The method for identifying appropriate publications is described in Fig. 5. Cases were indexed by age, symptoms, location of aneurysm or aSDH, treatment, examination, and outcome. Of the 8 cases, 3 were men and 5 were women. The age of patients ranged from 25 to 51 years. In 2 cases, the aSDH was located posterior to the communicating artery aneurysm. Three cases involved middle cerebral artery aneurysm, and 2 cases had an internal distal carotid artery aneurysm. Finally, 1 case had an aneurysm in the distal anterior cerebral artery. In these cases, headache was the most common initial symptom. However, coma, vomiting, and nausea also have been reported as presenting symptoms. Digital subtraction angiography (DSA) is the preferred diagnostic modality for aneurysms. Most of the retrieved cases were diagnosed by DSA. Three cases with aneurysm were not diagnosed immediately. One was diagnosed by indocyanine green videoangiography during aneurysm surgery after a diagnosis could not be made based on MRA and CTA. Hematoma evacuation and aneurysm clipping were performed for the treatment of all patients in the included studies. Good recovery was reported for 6 patients, while a poor recovery with disability was experienced by 2 patients. No deaths occurred among the 8 reviewed cases. The non-traumatic causes of aSDH are listed in Table 2.

**Table 1:**
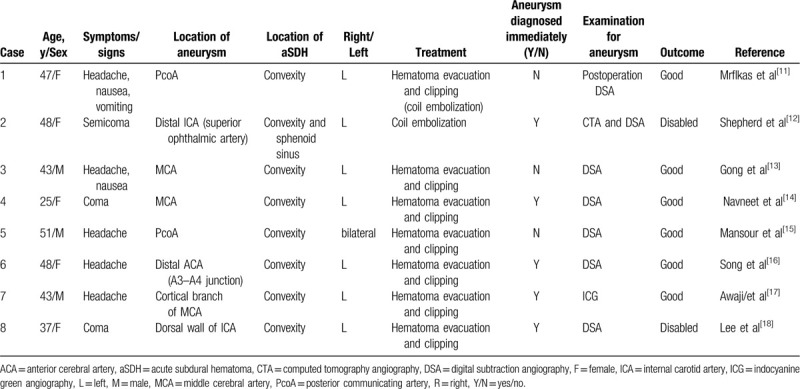
Summary of literature review: cases of non-traumatic aSDH caused by aneurysm.

**Table 2:**
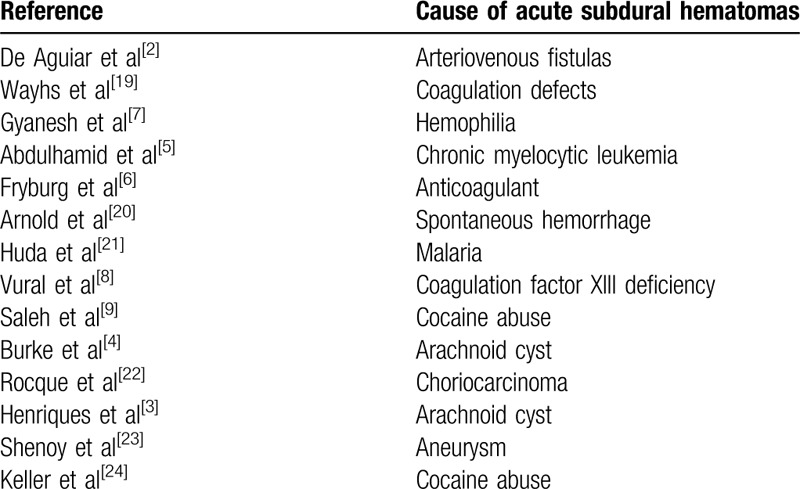
Acute subdural hematomas caused by non-traumatic reasons.

Few publications have addressed the prevalence of spontaneous non-traumatic aSDH, and spontaneous aSDH caused by aneurysm is quite rare.^[1]^ Aneurysms commonly lead to subarachnoid hemorrhage, ventricular hemorrhage, or intracranial hematoma, while trauma is the primary cause for aSDH.^[25]^ It is easy to overlook the existence of aneurysms in aSDH. Ohkuma et al^[1]^ reported that the incidence of subarachnoid hemorrhage or ventricular hemorrhage caused by aneurysm is only 1.5% to 2.7%, whereas the incidence of spontaneous aSDH caused by aneurysm was even lower due to the invisibility. With an aSDH, blood usually accumulates in the surface of dura mater and cerebral cortex, which may result in severe consequences due to compression.^[10]^

The cause of aSDH in the case reported here was confirmed to be aneurysm rupture. The pathogenesis of aSDH caused by aneurysm rupture remains unclear for now, but there are a few possible hypotheses. It was suggested that the high blood pressure created during aneurysm rupture may lead to aSDH as a result of damage to the arachnoid. Alternatively, the aSDH could be caused by the rupture of an aneurysm located in the subdural space.^[26]^ Ultimately, the normal structure of the arachnoid had been destroyed by a previous microvascular aneurysm hemorrhage, allowing the broken tissue from the aneurysm to penetrate the subdural space when a second rupture of the aneurysm occurred, which may likely be the pathogenesis of the aSDH in this case. The patient complained of a sudden headache twice in approximately 6 weeks. Although it was not seen on CT and MRI examinations, there is a high possibility that a microvascular aneurysm hemorrhage existed.^[27]^ Arachnoid adhesion and damage may also exist in this situation secondary to microvascular aneurysm hemorrhage, which was confirmed during the surgery. However, the second hypothesis cannot be completely ruled out, and the occurrence of aSDH in this case may have been influenced by a variety of interacting factors.

The definition of aSDH caused by aneurysm remains controversial. Spontaneous aSDH was suggested to represent aSDH caused by aneurysm, while a contrary opinion stated that spontaneous aSDH should be referred to as aSDH caused by spontaneous rupture of vasculature. Neutrally, spontaneous aSDH may include all forms of aSDH caused by non-traumatic reasons.^[2–9,19,21,22,23,28,29]^ There is a risk of missed diagnosis for aSDH caused by aneurysm. DSA has been considered the golden standard for diagnosing aneurysm, as it is capable of tracking the location of an aneurysm and identifying the relationship between the aneurysm and nearby artery. However, there is a risk of radiocontrast agent leakage during DSA procedures. Compared with DSA, CTA has the advantage of a shorter diagnostic time frame, which is especially beneficial for critically ill patients.^[30]^ Treatments options for aSDH include surgical decompression, hematoma evacuation, ventricular drainage, and conservative therapy. Conservative therapy is reasonable for minor defects. Ventricular drainage can be used in the absence of coagulation defects. However, surgical decompression and hematoma evacuation are advised to prevent adverse outcomes such as motor or sensory deficits.

Although a rare cause of aSDH, aneurysm should be considered in efforts to diagnose the cause of non-traumatic spontaneous aSDH. It is necessary to track the origin of bleeding while the patient is in relatively stable condition. DSA examination should be considered for patients diagnosed with an aSDH with unknown cause.

## Author contributions

**Conceptualization:** Yang Zhang, Wei Wang.

**Formal analysis:** Fagui Yue.

**Investigation:** Yang Sun.

**Methodology:** Fenglei Zhang, Yang Zhang, Xiaobo Zhu.

**Resources:** Xiaobo Zhu.

**Software:** Fenglei Zhang.

**Supervision:** Wei Wang.

**Validation:** Yang Sun.

**Visualization:** Fenglei Zhang.

**Writing – original draft:** Xianfeng Gao, Fagui Yue.

**Writing – review & editing:** Wei Wang.

## References

[1] Ohkuma H Shimamura N Fujita S Acute subdural hematoma caused by aneurysmal rupture: incidence and clinical features. Cerebrovasc Dis 2003;16:171–3. 12792176 10.1159/000070598

[2] de Aguiar GB Veiga JC Silva JM Spontaneous acute subdural hematoma: a rare presentation of a dural intracranial fistula. J Clin Neurosci 2016;25:159–60. 26541324 10.1016/j.jocn.2015.05.057

[3] Henriques JG Pianetti Filho G Henriques KS Spontaneous acute subdural hematoma contralateral to an arachnoid cyst. Arq Neuropsiquiatr 2007;65:1034–6. 18094872 10.1590/s0004-282x2007000600025

[4] Burke MP O’Donnell C Opeskin K Spontaneous acute subdural hematoma complicating arachnoid cyst. Am J Forensic Med Pathol 2010;31:382–4. 21063200 10.1097/PAF.0b013e3181fc354b

[5] Abdulhamid MM Li YM Hall WA Spontaneous acute subdural hematoma as the initial manifestation of chronic myeloid leukemia. J Neurooncol 2011;101:513–6. 20582615 10.1007/s11060-010-0278-6

[6] Fryburg K Nguyen HS Cohen-Gadol AA Spontaneous acute subdural hematoma due to fondaparinux: report of two cases. Surg Neurol Int 2011;2:44. 21660271 10.4103/2152-7806.79759 PMC3108447

[7] Gyanesh P Dhiraaj S Anesthetic management of a patient with hemophilia A with spontaneous acute subdural hematoma. J Anaesthesiol Clin Pharmacol 2013;29:117–20. 23494075 10.4103/0970-9185.105819 PMC3590516

[8] Vural M Yarar C Durmaz R Spontaneous acute subdural hematoma and chronic epidural hematoma in a child with F XIII deficiency. J Emerg Med 2010;38:25–9. 18514462 10.1016/j.jemermed.2007.11.041

[9] Saleh T Badshah A Afzal K Spontaneous acute subdural hematoma secondary to cocaine abuse. South Med J 2010;103:714–5. 20531038 10.1097/SMJ.0b013e3181d7e378

[10] Danish SF Burnett MG Ong JG Prophylaxis for deep venous thrombosis in craniotomy patients: a decision analysis. Neurosurgery 2005;56:1286–92. discussion 1292-4. 15918945 10.1227/01.neu.0000159882.11635.ea

[11] Mrfka M Pistracher K Augustin M Acute subdural hematoma without subarachnoid hemorrhage or intraparenchymal hematoma caused by rupture of a posterior communicating artery aneurysm: case report and review of the literature. J Emerg Med 2013;44:e369–73. 23561314 10.1016/j.jemermed.2012.11.073

[12] Shepherd D Kapurch J Datar S Sphenoid and subdural hemorrhage as a presenting sign of ruptured clinoid aneurysm. Neurocrit Care 2014;20:489–93. 23893075 10.1007/s12028-013-9866-6

[13] Gong J Sun H Shi XY Pure subdural haematoma caused by rupture of middle cerebral artery aneurysm: case report and literature review. J Int Med Res 2014;42:870–8. 24691457 10.1177/0300060514524929

[14] Singla N Tripathi M Chhabra R M5 segment aneurysm presenting as “pure acute SDH”. J Neurosci Rural Pract 2014;5:402–4. 25288848 10.4103/0976-3147.140002 PMC4173243

[15] Mansour O Hassen T Fathy S Acute aneurismal bilateral subdural haematoma without subarachnoid haemorrhage: a case report and review of the literature. Case Rep Neurol Med 2014;2014:260853. 25045554 10.1155/2014/260853 PMC4086224

[16] Song TW Kim SH Jung SH Rupture of distal anterior cerebral artery aneurysm presenting only subdural hemorrhage without subarachnoid hemorrhage: a case report. Springerplus 2016;5:73. 26844020 10.1186/s40064-016-1727-2 PMC4726640

[17] Awaji K Inokuchi R Ikeda R Nontraumatic pure acute subdural hematoma caused by a ruptured cortical middle cerebral artery aneurysm: case report and literature review. NMC Case Rep J 2016;3:63–6. 28664000 10.2176/nmccrj.cr.2015-0151 PMC5386168

[18] Lee YW Nam TM Kim JS Pure subdural hemorrhage caused by internal carotid artery dorsal wall aneurysm rupture. J Cerebrovasc Endovasc Neurosurg 2016;18:302–5. 27847779 10.7461/jcen.2016.18.3.302 PMC5104860

[19] Wayhs SY Wottrich J Uggeri DP Spontaneous acute subdural hematoma and intracerebral hemorrhage in a patient with thrombotic microangiopathy during pregnancy. Rev Bras Ter Intensiva 2013;25:175–80. 23917984 10.5935/0103-507X.20130030 PMC4031834

[20] Aldawood A Clinical characteristics and outcomes of critically ill obstetric patients: a ten-year review. Ann Saudi Med 2011;31:518–22. 21911991 10.4103/0256-4947.84631 PMC3183688

[21] Huda MF Kamali NI Srivastava VK Spontaneous acute subdural hematoma in malaria: a case report. J Vector Borne Dis 2011;48:247–8. 22297290

[22] Rocque BG Başkaya MK Spontaneous acute subdural hematoma as an initial presentation of choriocarcinoma: a case report. J Med Case Rep 2008;2:211. 18565226 10.1186/1752-1947-2-211 PMC2442118

[23] Shenoy SN Kumar MG Raja A Intracranial aneurysms causing spontaneous acute subdural hematoma. Neurol India 2003;51:422–4. 14652465

[24] Keller TM Chappell ET Spontaneous acute subdural hematoma precipitated by cocaine abuse: case report. Surg Neurol 1997;47:12–4. discussion 14-5. 8986158 10.1016/s0090-3019(96)00380-1

[25] Vega RA Valadka AB Natural history of acute subdural hematoma. Neurosurg Clin N Am 2017;28:247–55. 28325459 10.1016/j.nec.2016.11.007

[26] Hatayama T Shima T Okada Y Ruptured distal anterior cerebral artery aneurysms presenting with acute subdural hematoma: report of two cases. No Shinkei Geka 1994;22:577–82. 8015681

[27] Cianfoni A Pravatà E De Blasi R Clinical presentation of cerebral aneurysms. Eur J Radiol 2013;82:1618–22. 23238357 10.1016/j.ejrad.2012.11.019

[28] Tatli M Guzel A Altinors N Spontaneous acute subdural hematoma following contralateral calcified chronic subdural hematoma surgery: an unusual case. Pediatr Neurosurg 2006;42:122–4. 16465084 10.1159/000090468

[29] Arnold PM Christiano LD Klemp JA Nontraumatic spontaneous acute subdural hematoma in identical teenage twins 1 year apart. Pediatr Emerg Care 2011;27:649–51. 21730803 10.1097/PEC.0b013e3182225637

[30] Saleem T Baril DT Baril, Aneurysm, Femoral, Repair, in StatPearls. Treasure Island, FL: StatPearls Publishing LLC; 2017.

